# Leptomeningeal disease: current diagnostic and therapeutic strategies

**DOI:** 10.18632/oncotarget.20272

**Published:** 2017-08-16

**Authors:** Gautam Nayar, Tiffany Ejikeme, Pakawat Chongsathidkiet, Aladine A. Elsamadicy, Kimberly L. Blackwell, Jeffrey M. Clarke, Shivanand P. Lad, Peter E. Fecci

**Affiliations:** ^1^ Duke Brain Tumor Immunotherapy Program, Department of Neurosurgery, Duke University Medical Center, Durham, NC, USA; ^2^ The Preston Robert Tisch Brain Tumor Center, Duke University Medical Center, Durham, NC, USA; ^3^ Department of Neurosurgery, Duke University Medical Center, Durham, NC, USA; ^4^ Department of Radiation Oncology, Duke University Medical Center, Durham, NC, USA; ^5^ Division of Medical Oncology, Duke University Medical Center, Durham, NC, USA; ^6^ Department of Pathology, Duke University Medical Center, Durham, NC, USA

**Keywords:** leptomeningeal disease, neoplastic meningitis, leptomeningeal carcinomatosis, intrathecal chemotherapy, radiation therapy

## Abstract

Leptomeningeal disease has become increasingly prevalent as novel therapeutic interventions extend the survival of cancer patients. Although a majority of leptomeningeal spread occurs secondary to breast cancer, lung cancer, and melanoma, a wide variety of malignancies have been reported as primary sources. Symptoms on presentation are equally diverse, often involving a combination of neurological deficits with the possibility of obstructive hydrocephalus. Diagnosis is definitively made via cerebrospinal fluid cytology for malignant cells, but neuro-imaging with high quality T1-weighted magnetic resonance imaging can aid diagnosis and localization. While leptomeningeal disease is still a terminal, late-stage complication, a variety of treatment modalities, such as intrathecal chemotherapeutics and radiation therapy, have improved median survival from 4–6 weeks to 3–6 months. Positive prognosticative factors for survival include younger age, high performance scores, and controlled systemic disease. In looking to the future, diagnostics that improve early detection and chemotherapeutics tailored to the primary malignancy will likely be the most significant advances in improving survival.

## INTRODUCTION

Leptomeningeal disease (LMD) is a late-stage complication of systemic cancers caused by multifocal metastases to the leptomeninges, which consist of the pia mater, arachnoid, and subarachnoid space. Common neoplastic etiologies of LMD include breast, lung (mainly non-small-cell lung cancer [NSCLC]), gastro-intestinal, melanoma, primary central nervous system (CNS) cancers (such as medulloblastoma, ependymoma, pineoblastoma, primitive neuroectodermal, or primary CNS lymphoma), lymphoma (mainly non-Hodgkin's Lymphoma [NHL]), leukemia (mainly acute lymphoblastic leukemia [ALL]), and multiple myeloma [1–3].

Despite advances in targeted radiation and chemotherapy, survival remains poor after diagnosis of leptomeningeal involvement, averaging 3–6 months. Survival varies by etiology, with breast cancer patients having the best prognosis (13–25% survival at one year and 6% at two years) [1, 4, 5]. Predictors of longer survival include younger age of diagnosis, a Karnofsky Performance Score (KPS) above 70, long duration of symptoms, controlled systemic disease, lack of encephalopathy or focal neurological deficits on examination, low levels of cerebrospinal fluid (CSF) protein, and lack of bulky disease on imaging [1, 6, 7].

## EPIDEMIOLOGY

In the United States, 1–8% of cancer patients are diagnosed with LMD, with approximately 110,000 cases of LMD per year in the U.S. [4] LMD is found in 1–5% of patients with solid tumors, and 1–2% of patients with primary brain tumors (Table 1). The exact incidence of LMD is difficult to determine, since gross examination at autopsy may overlook signs of LMD, and microscopic pathological inspection may be normal if the seeding is multifocal or if an unaffected area of the CNS is examined. Adenocarcinomas are the most common tumors to metastasize to the leptomeninges. Of the patients with NSCLC, 30–64% have CNS metastases, of which 4–7% have LMD [5, 8, 9].

**Table 1:** Common LMD etiologies

Primary-Specific Characteristics of LMD			
Primary Cancer	Prevalence of LMD	Prognosis	Additional Treatment Modalities
Melanoma	30–75%	Median: 6.9 months	BRAF inhibitors (Vemurafenib and Dabrafenib) and Checkpoint inhibitors (Ipilimumab and Nivolumab) improved survival (16.9 weeks vs. 2.9 weeks) in prospective studies
NHL	5–30%	Median: 2.6 months	IT Rituxumab in Phase 1 studies. Prophylaxis with IT chemotherapy.
NSCLC	9–25%	Median: 3.5 months	EGFR TKI improved survival if EGFR+ in multiple case reports
Breast Cancer	5%	Median: 4.2 months (longer if hormone receptor positive)	High-Dose MTX trial (NCT02422641) pending. IT Trastuzumab if HER2+.

Incidence and prevalence of LMD are both increasing due to better imaging modalities and improved ability to treat, such as with epidermal growth factor receptor (EGFR) tyrosine kinase inhibitors, anaplastic lymphoma kinase (ALK) inhibitors, and whole brain radiation. EGFR mutations are seen in 10–15% of Caucasians and 30–40% of Asian NSCLC cases. ALK gene rearrangements are found in 4–5% of NSCLC [10]. Overall, 9–25% of patients with small-cell lung cancers demonstrated LMD. CNS involvement is seen clinically in 30% of patients with melanoma, and as high as 75% at autopsy [11]. Although only 5% of patients with breast cancer develop leptomeningeal involvement, it remains the most common etiology of LMD [12]. The second most common tumor with LMD is lung cancer. Rare neoplasms, such as retinoblastoma and embryonal rhabdomyosarcoma, can also spread to leptomeninges. Occasionally, LMD originating from sarcomas can also be found, although it is uncommon. Medulloblastomas, ependymomas, and gliomas are tumors of intracranial origin that can spread to the CSF and elicit LMD. However, LMD remains rare in high-grade gliomas due to rapid deterioration that precludes leptomeningeal involvement [13]. Squamous cell carcinomas of the head and neck likewise can spread to the leptomeninges along the cranial nerve tracts. Intracranial metastases accompanied LMD in 98% of patients with a non-leukemic primary cancer [14].

LMD has also become more frequent with as survival has extended for cancer patients [15]. The longer a patient bears his or her primary cancer, the higher the prevalence of LMD, as control of non-CNS cancers may allow for more time for the development of CNS metastases. Additionally, the CNS may be a particular repository for some cancer subtypes and/or treatment exposures. For instance, ALK gene arrangements characterizing a subtype of NSCLC are normally responsive to crizotinib, but patients treated with this agent are frequently found with intracranial metastases at relapse [16]. As such, use of large-molecule anti-neoplastic agents with limited CNS and CSF penetration may control systemic disease, but leave LMD untreated behind the blood-brain and blood-CSF barrier.

## CLINICAL FEATURES

As malignant cells can spread to any area of the CNS and precipitate symptoms, leptomeningeal disease can have various initial clinical presentations. Common symptoms include cranial nerve deficits, radicular pain, headache, back pain, visual disturbances, diplopia, hearing loss, onset of psychiatric disorders, seizures, or cauda equina syndrome [1, 5, 17–19]. Proposed mechanisms leading to these symptoms include direct compression of parenchyma, parenchymal invasion, ischemia secondary to vessel involvement, metabolic strain, and disruption of the blood-brain barrier [20]. Furthermore, malignant growth can impair flow of CSF in more than half of patients, leading to symptoms of obstructive or communicative hydrocephalus such as nausea, vomiting, somnolence, and positional headaches.

Findings may initially be subtle, such as isolated diplopia or radicular pain, and so are prone to being dismissed in patients who are sick with metastatic disease. However, symptoms progress quickly in severity and evolve along multiple segments of the neuraxis [21]. The development of such neurological symptoms in a patient with known metastatic disease is highly suspicious for leptomeningeal involvement [22]. Nevertheless, it remains important to rule out alternative causes, such as parenchymal disease, chemotherapy or radiation side effects, paraneoplastic syndromes, sarcoidosis, or infectious etiologies [23].

## DIAGNOSTICS

### Cerebrospinal fluid

Presence of malignant cells on CSF cytology provides the gold-standard for diagnosing leptomeningeal carcinomatosis [24]. If location of the disease is suggested by symptomatology, fluid should be drawn from nearby. Otherwise, fluid should be drawn from the lumbar or cisternal regions, as this has shown increased sensitivity and specificity compared to intraventricular fluid [1]. While the first lumbar puncture is only 50–60% sensitive, a repeat collection increases sensitivity to 80%. Additional lumbar punctures increase the sensitivity by 2–5% per collection. Each CSF collection should draw 5–10 mL to ensure a sufficient amount for analysis [25].

If malignant cells are not seen, analysis of CSF protein levels can be informative [1, 17]. Particularly, a protein level above 45 mg/dL is seen in 63–90% of patients with leptomeningeal disease. If levels are very high (> 500 mg/dL), then there is likely blockage or advanced disease. Another useful predictor of carcinomatous pathology is high CSF pressure (> 150 mm), seen in 30–57% of patients. Furthermore, CSF pleocytosis is seen in 33–79% of patients with leptomeningeal disease, and glucose levels are often decreased (< 60 mg/dL) in 24–62% of patients [26]. Conversely, a normal CSF profile is seen in less than 5% of patients with leptomeningeal disease, and is therefore a strong negative predictor.

Methods for CSF analysis specific to the primary malignancy, such as immuno-histochemistry, flow cytometry, fluorescent *in-situ* hybridization, or polymerase chain reaction, can further improve detection rates [27–29]. Furthermore, CSF analysis for common tumor-specific antigens, such as CA 15-3 or carcinoembryonic antigen (CEA), can be considered if the primary malignancy is known [30–33]. An early study has shown carcinoembryonic antigen to be synthesized intrathecally in 89% of meningeal carcinomas, but also in 47% of intraparenchymal carcinomas, which may lead to misdiagnosis [34]. CSF levels of the proangiogenic factor, VEGF, when standardized to serum albumin and VEGF levels, have been shown to produce a relatively sensitive (83.3%) and specific (88.4%) method of diagnosis for LMD [35]. Adjustment for serum levels is important given that leptomeningeal disease can damage the blood-brain barrier and so alter diffusion [26]. Given these drawbacks and a lack of quality studies, tumor-specific antigens are currently not included in any standardized diagnostic algorithm. Given the variety of sensitive analytical methods, CSF studies continue to provide the most definitive approach to diagnosis of leptomeningeal disease.

### Neuro-radiography

Abnormalities on imaging can be found in 70–80% of patients with leptomeningeal disease, although more often in solid versus hematological primary cancers [1, 17, 36]. The imaging modality of choice is high quality, T1-weighted magnetic resonance imaging (MRI) with gadolinium contrast, which has been shown to be more sensitive compared to contrast enhanced CT [37, 38]. All imaging should include the brain and spine, as leptomeningeal disease can impact the entire neuraxis.

On MRI, the most common finding is pial enhancement and nodularity, typically over the cerebral convexities, in the basal cisterns, on the tentorium, or in the ventricular ependymal surfaces [26, 38]. Common findings on imaging of the spinal cord include patchy involvement of nerve roots with occasional matting and intradural extramedullary nodules, particularly at the cauda equina [39]. This is particularly important in allowing localization of symptomatic lesions for palliative treatment, such as those causing painful radiculopathy or obstructive hydrocephalus.

Despite advances in imaging techniques and development of novel MRI sequences over the past few decades, early detection of leptomeningeal disease still has not been found to impact overall survival [2]. Furthermore, these findings are fairly non-specific, and could be seen in alternative etiologies such as meningitis or lumbar puncture induced intracranial hypotension [40]. Clinical context remains key, and suspicious findings should prompt further analysis in patients with malignancies.

### CSF flow studies

Obstructed flow of cerebrospinal fluid (CSF) develops in 30–70% of patients with leptomeningeal carcinomatosis [1, 41]. Compared to conventional MRI, CSF flow studies that utilize Indium-111 DTPA or Technetium-99m labeled albumin are more sensitive for and can better characterize the location of obstructive hydrocephalus, allowing for palliative intervention with focused radiotherapy [42]. Furthermore, failed therapy for the obstructive lesion is a strong predictor for rapid neurological decline and death [1, 43]. CSF flow studies are used prior to initiation of intrathecal chemotherapy, to assess for regions of poor drug penetration or toxic drug accumulation. However, routine analysis of CSF flow for prognostic evaluation remains a rare practice among clinicians.

### Diagnostic approach

The majority of diagnostic algorithms, including recent criteria developed by the Response Assessment in Neuro-Oncology (RANO) group with expertise in LMD, recommend initial evaluation with a CSF profile, CSF cytology, and high quality, gadolinium enhanced MRI [7, 26, 33]. Positive CSF cytology and radiographic evidence is enough to make the diagnosis. A negative initial evaluation should be followed by at least one additional lumbar puncture. However, it has been estimated that up to 25% of patients with symptomatic LMD can have negative diagnostic evaluations. It remains controversial whether these patients should be presumptively treated. In rare cases, biopsy of the leptomeninges or brain can provide an accurate diagnosis, especially if the primary malignancy is unknown [17].

## BIOLOGICAL BASIS

### Strategies for spread

The leptomeninges, as a component of the CNS, are more resistant to metastatic disease due to the blood-brain barrier and the blood-CSF barrier. However, the mechanism by which malignant cells bypass these barriers are largely unknown and may vary between primary malignancies. Understanding this process could lead to the development of novel therapeutics that interfere with these mechanisms [44].

Malignant cell seeding can occur in many ways, including hematogenous spread, venous dissemination, or direct invasion. One possible avenue of entry into the CNS could involve the fenestrated endothelium of the choroid plexus, which allows selective passage of solutes otherwise prohibited by intact barriers and tight junctions [45]. Subsequently, spread after initial leptomeningeal involvement is facilitated by constant CSF flow and regulated by various cytokines and growth factors.

Among these inflammatory cytokines is VEGF, a key participant in tumor angiogenesis and endothelial cell proliferation. Previous studies have shown VEGF to be an important biomarker for malignant cells in the CSF by inducing transendothelial migration in breast cancer cells. Its elevated CSF levels are very sensitive and specific for LMD diagnosis from breast cancer, lung cancer, and melanoma, making VEGF an important drug target [46–48]. For instance, Bevacizumab is a monoclonal antibody directed against VEGF that can be administered systemically for patients with LMD.

Chemokines in the CSF are responsible for regulating proliferation, trafficking, and adhesion of leukocytes. Cells with elevated metastatic potential to the CNS are likely to express the chemokine CXCR4 and its ligand stromal cell-derived factor (SDF)-1. CXCR4/SDF-1 signaling has been shown to induce vascular permeability as well as tumor cell migration and penetration through brain microvascular endothelial cells. Additional chemokines such as CXCR1, CXCR2, and CXCL-8 receptors are often overexpressed in melanoma and promote tumor growth and invasion [49–51].

## THERAPY

### Current therapy

**Figure 1:**
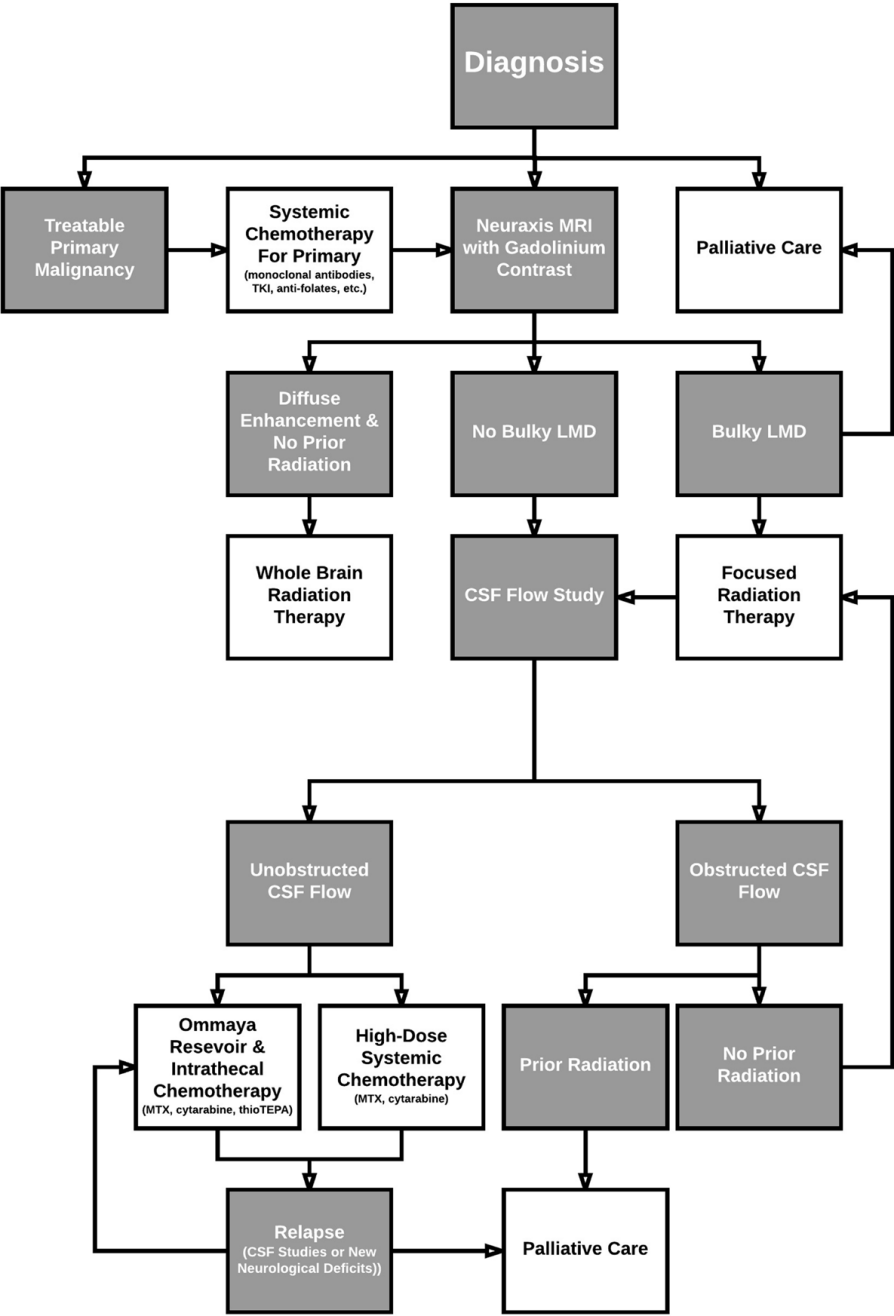
Treatment algorithm

Leptomeningeal disease is difficult to treat, with generally poor outcomes. Primary treatment goals include improvement of patients’ neurological deficits and quality of life, while avoiding toxicity. Patient selection factors for treatment include high KPS scores and younger age [1]. While one approach to treatment is shown in Figure 1, a lack of large randomized controlled studies has made the choice of therapy controversial [21]. Common therapeutic approaches include radiation therapy to symptomatic anatomical locations and sites where neuroimaging has revealed lesions, followed by intrathecal chemotherapy [52]. Radiation at the local site of lesion is mainly used to alleviate neurological symptoms, primarily by reducing bulky disease to increase chemotherapeutic perfusion to areas with poor flow. Intrathecal chemotherapy can then reduce tumor cells in the CSF and leptomeningeal deposits, preventing additional seeding [53, 54]. Systemic chemotherapy can be added to the treatment regimen to further treat primary tumors and prolong survival (Table 2) [52]. For example, in a retrospective study of 30 patients with NSCLC complicated by LMD, Riess *et al.* found that receiving a systemic regimen containing pemetrexed, bevacizumab, or a tyrosine kinase inhibitor demonstrated a statistically significant decreased hazard of death (hazard ratio [HR], 0.24; *p*-value = 0.007) [55]. In addition to disease management, symptomatic treatments include anxiolytics, antidepressants, pain controls, and anticonvulsants.

**Table 2:** Clinical trials

Identifier	Acronym	Phase	Number of Patients	Therapy Class	Therapy Agent	Dosing Schedule	Primary (P)/Secondary (S) Endpoints	Results
NCT01283516	ASCEND1	I	246	Tyrosine Kinase Inhibitor	Ceritinib	750 mg daily	P: Dose Limiting Toxicities (DLT); Overall Response Rate (ORR); Duration of Response (DOR) S: Drug Related Adverse Events; Absorption and Plasma Concentration of LDK378	DLT: 8 Pts ORR: 56.4% (All Pts) Median DOR: 7.39 mos (All Pts) Median PFS: 18.4 mos – ALK Pretreated Most Common AE: Elevation of AST/ALT Levels
NCT01685060	ASCEND2	II	140	Tyrosine Kinase Inhibitor	Ceritinib	750 mg daily	P: ORR S: DOR; Disease Control Rate (DCR); Time to Response (TTR); Safety Profile; PFS; OS; Overall Intracranial Response Rate (OIRR)	ORR: 38.6% (All Pts) DCR: 77.1% (All Pts) Median DOR: 9.7 mos (All Pts) Median PFS: 9.7 mos (All Pts) Most Common AE: Nausea, Diarrhea, Vomiting
NCT01685138	ASCEND3	II	124	Tyrosine Kinase Inhibitor	Ceritinib	750 mg daily	P: ORR S: DOR; Disease Control Rate (DCR); Time to Response (TTR); Safety Profile; PFS; OS; Overall Intracranial Response Rate (OIRR)	ORR: 63.7% (All Pts) DCR: 89.5% (All Pts) Median DOR: 9.3 mos (All Pts) Median PFS: 11.1 mos (All Pts) Most Common AE: Diarrhea, Nausea, Vomiting
NCT01828099	ASCEND4	III	376	Tyrosine Kinase Inhibitor	Ceritinib	750 mg daily	P: PFS S: OS; ORR; DOR; DCR: TTR	Median PFS: Ceritinib (16.5 mos) vs Chemo (8.1 mos) OS: Ceritinib (29.3 mos) vs Chemo (26.2 mos) ORR: 72.5% (Ceritinib) DCR: 84.7% (Ceritinib) DOR: 23.9 mos (Ceritinib) Most Common AE: Diarrhea, Nausea, Vomiting
NCT01828112	ASCEND5	III	231	Tyrosine Kinase Inhibitor	Ceritinib	750 mg daily	P: PFS S: OS; ORR; DOR; DCR: TTR	Median PFS: Ceritinib (5.4 mos) vs Chemo (1.6 mos) ORR: 39.1% (Ceritinib) DCR: 76.5% (Ceritinib) Most Common AE: Diarrhea, Nausea, Vomiting
NCT02616393		II	60	Tyrosine Kinase Inhibitor	Tesevatinib		P: Clinical Activity Against BM and LM S: Quality of Life Assessments; PFS; OS	
		II	19	Folate Antimetabolite	Temozolomide	One cycle of oral TMZ (100 mg/m(2) daily) one week on treatment/one week off treatment for four weeks.		Study stopped early due to poor accrual. 3 of 19 Pts demonstrated clinical benefit.
NCT00424242		I	15	Folate Antimetabolite	Pemetrexed		P: Correlation of CSF with Plasma Levels of Different Doses; Anti-Tumor activity against LM; Safety Profile; Assess role of Serum Biomarkers in Pts with LM	
NCT01281696		I/II	8	Monoclonal Antibody	Bevacizumab	Bevacizumab (Day 1), Etoposide (Days 2-4), and Cisplatin on Day 2 in a 21-day cycle for 6 cycles.	P: CNS Response Rate	CNS Response Rate: 60% in 5 evaluable pts. Median OS: 4.7 mos Neurologic PFS: 4.7 mos Most Common AE: Neutropenia, Leukopenia, Hyponatremia
NCT01325207		I/II	34	Monoclonal Antibody	Trastuzamab	10–500 mg Twice Weekly	P: Safety and maximum tolerated dose of intrathecal (IT) Trastuzumab S: Response to IT Trastuzumab; CSF Pharmokinetic of IT Trastuzumab	

### Radiation

Radiation therapy is an important modality for providing palliative relief to patients with symptomatic leptomeningeal disease. Cranio-spinal radiation is typically avoided, as marrow toxicity compromises the patient's ability to undergo future chemotherapeutic regimens. Patients with cerebral involvement typically receive whole brain radiotherapy (WBRT), whereas those with symptomatic spinal lesions are candidates for focal radiotherapy. WBRT is usually planned to involve all neural tissue from the retro-orbit to the upper cervical vertebrae. Radiation is usually administered at a dose of 3 Gy for 10 days, but can vary between 20–40 Gy as patients with better prognosis tend to receive more sessions. However, there has not been a documented benefit in survival from WBRT [4, 56–58]. Furthermore, WBRT can cause transient somnolence and cognitive decline. On the other hand, focal radiotherapy is well-proven in providing palliative relief, such as reduction of radiculopathies, bulky disease, or obstructive lesions causing hydrocephalus [59]. Resolution of obstructive lesions also increases penetrance and distribution of intra-thecal chemotherapeutics, and so may precede chemotherapy [58].

### Intra-thecal chemotherapy

Intrathecal administration is the most common method to deliver chemotherapeutic agents in non-nodular and non-bulky LMD, although efficacy compared to systemic administration and choice of regimen are poorly understood due to limited randomized controlled trials [21]. Chemotherapies are usually hydrophilic, and therefore do not penetrate the blood-brain barrier well (< 5%) [20]. Methotrexate (MTX), cytarabine (Ara-C), and thiotepa are commonly administered intrathecally for LMD. Methotrexate is the most studied agent, with standard therapy consisting of two cycles of 10–15 mg twice weekly for 4–6 weeks, followed by monthly maintenance therapy with 10–15 mg if cytological clearance is achieved [60]. Sustained release formulations may offer increased therapeutic value, as a randomized controlled trial of 61 patients with LMD comparing the efficacy of IT sustained-release cytarabine to methotrexate found similar efficacy but significantly increased time to neurological progression and easier-to-manage administration schedule for sustained-release cytarabine [61, 62]. Several case reports have also demonstrated decreased progression and improved prognosis of LMD from breast cancer following intrathecal trastuzumab, a monoclonal antibody that targets malignant cells that overexpress HER2 [63–66].

### Surgical interventions

Surgical interventions include ventriculo-peritoneal shunting and placement of intraventricular catheters. Shunting is used in the setting of obstructive hydrocephalus secondary to bulky LMD [20]. Catheter placement allows for the delivery of intra-thecal chemotherapy from a subgaleal reservoir (eg. Ommaya), which increases ease of access while providing more uniform drug distribution and more reliable delivery compared to repeated lumbar administration [17, 33, 67]. However, the complication rate remains high, particularly due to catheter failure and infection. Furthermore, no substantial benefits for survival have been seen with this approach [1, 68].

### Systemic chemotherapy

Based on numerous reports, systemic treatments for LMD are believed to increase patient survival. In fact, some authors consider it to be the more vital component to treatment, and thereby exclude intrathecal therapies altogether in patients with responsive cancers. Systemic treatment allows for a significant reduction in toxicity following administration versus intrathecal treatment, while outcomes remain similar [69, 70].

### Methotrexate

**Figure 2:**
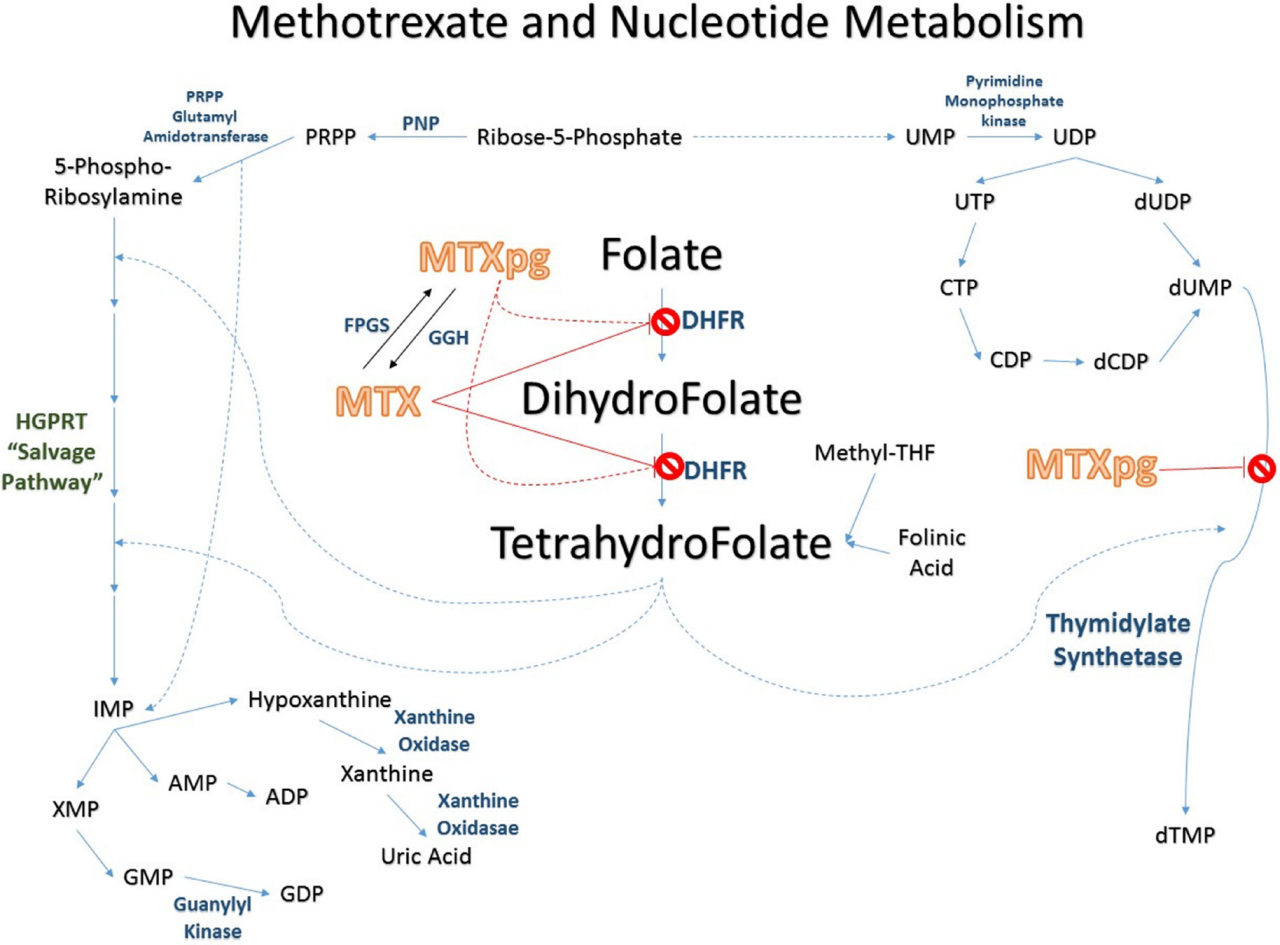
Methotrexate pathway

Methotrexate is an inhibitor or dihydrofolate reductase (DHFR), thymidylate synthetase, and many other NAD(P)H-dependent oxidoreductases (Figure 2). By interfering with the purine synthesis pathway and DNA repair, methotrexate is able to regulate cancer cell metabolism. Methotrexate has a high CSF penetration at high doses. A prospective, nonrandomized study compared the results of bolus intrathecal (*n* = 15, two doses per week for 4 weeks) versus high-dose (*n* = 16, 8 g/m^2^ over 4 hours for 2–4 sessions) systemic methotrexate administration in patients with leptomeningeal disease. High-dose systemic methotrexate (8 g/m^2^ over 4 hours) showed a higher CSF tumor cell clearance and longer patient survival (13.8 months versus 2.3 months, *P* = 0.03) compared to bolus intrathecal methotrexate [71].

Though proven to be beneficial at high doses, intracellular metabolism of methotrexate yields an accumulation of its poly-glutamated form, which is known to be a source of toxicity. Leucovorin, also known as folinic acid, is typically given with methotrexate to rescue cells from any adverse effects without affecting the DHFR blockade [72, 73].

### Capecitabine

Capecitabine, a chemotherapy whose metabolite can inhibit *de novo* DNA synthesis, is used to treat gastric, colorectal, and breast cancer [74–76]. While randomized controlled trials are lacking, a retrospective analysis of three patients with refractory LMD originating from breast cancer by Ekenel *et al*. found that addition of capecitabine provided symptomatic relief and may have improved survival [77]. Another study examining two cases of LMD from breast cancer and one case of LMD from esophageal cancer suggested benefits of oral capecitabine administration [78]. Similarly, a case report documents a patient who had no neurological deficits or symptoms and had her LMD cleared on neuroimaging studies after 3.7 years of oral capecitabine monotherapy following WBRT, although she had persistent periodic presence of malignant cells in her CSF [79]. Further study with randomized control trials is required to understand the efficacy of capecitabine in patients with LMD.

### Temozolamide

Temozolamide (TMZ), a DNA alkylating agent, is an established chemotherapy and the standard of care along with radiotherapy for patients with malignant glioma [80, 81]. While case reports suggest possible efficacy against LMD secondary to glioma, a randomized control trial of 31 patients by Broniscer *et al*. showed no impact on outcome [82–86]. Similarly, case reports have suggested efficacy against LMD secondary to paragangliomas [87], adenocarcinomas [88–90], SCLC [91], breast cancer [62], and melanoma [92–94]. However, a phase II randomized controlled trial of 19 patients with LMD from solid tumors was prematurely stopped due to poor accrual, with median time to progression of 28 days (95% CI: 14–42 days), median survival of 43 days (95% CI: 28.7–57.3 days), and only two patients finishing the trial [95].

### Pemetrexed

Pemetrexed is a newer generation multi-targeted anti-folate agent, which is approved for the treatment of NSCLC but has been shown to have broader anti-tumor activity. Compared to methotrexate, pemetrexed requires no inpatient hospitalization, has better tolerability, exhibits a more favorable side effect profile, and possesses fewer drug-drug interactions [96]. Furthermore, common toxicities such as myelosuppression and gastro-intestinal disturbances can be predicted prior to therapy through analysis of homocysteine and methyl-malonic acid levels and prevented with vitamin supplementation [97]. As with methotrexate, CNS penetration of the drug is poor, with peak distribution reaching < 5% of plasma concentrations within one to four hours, and therefore has shown limited efficacy or survival benefit in leptomeningeal disease [96]. Intrathecal administration has not been well explored, with a single animal study finding that intrathecal pemetrexed distributed well and had significant half-life at 1mg/kg dosing [98].

One ongoing Phase 1 trial (NCT00424242) exploring intrathecal pemetrexed, being conducted at Northwestern University, is enrolling 15 adult patients with LMD. Escalating dosages of pemetrexed beginning at 500 mg/m^2^ are administered every 21 days until disease progression. Blood and CSF values are obtained to evaluate drug levels in both compartments. The levels of vascular endothelial growth factor and YKL 40 are assessed as markers of prognosis and response.

### Bevacizumab

Bevacizumab (Avastin) is an anti-VEGF monoclonal antibody commonly used for leptomeningeal enhancement from NSCLC and breast cancer, in which VEGF is significantly elevated and can indicate poor prognosis [46, 48, 99, 100]. Furthermore, recent reports have shown that bevacizumab can improve intra-tumor penetration of other chemotherapeutic agents, such as etoposide and cisplatin in breast cancer [101, 102], carboplatin and paclitaxel in NSCLC [55, 103], and erlotinib in EGFR positive cancers [104], perhaps by normalizing angiogenesis at the tumor site. Bevacizumab, along with temozolamide, has also shown efficacy against leptomeningeal dissemination of recurrent glioblastoma [86]. However, the safety profile of bevacizumab is dependent upon excluding and treating brain metastases due to risk of CNS hemorrhage at the tumor site [103, 105]. Additionally, initial case series of patients on bevacizumab report minimized radiological evidence of LMD, increased multifocal tumor spread, and dementia secondary to vasculopathy [106, 107].

A recent example is a Phase II trial of 39 patients with LMD secondary to breast cancer. Enrolled patients received bevacizumab plus etoposide and cisplatin (BEEP) every three weeks for a maximum of six cycles. The primary endpoints were the clearance of cancer cells in the CSF and improved neurological symptoms. The effect of bevacizumab on etoposide delivery into the CSF was also assessed. The median overall survival of eight enrolled patients was 4.7 months. In five evaluable patients, three exhibited clearance of cancer cells in the CSF. The neurologic progression-free survival was 4.7 months. Hyponatremia, leukopenia, and neutropenia were reported as the most commons adverse events. Bevacizumab did not impact etoposide penetration into the CSF, and etoposide concentrations in the CSF were remarkably lower than those in blood [101].

### Tyrosine kinase inhibitors

Tyrosine kinase inhibitors (TKIs) such as erlotinib, gefetinib, and afatinib have proven efficacy in EGFR-mutated NSCLC [108, 109]. Initial studies suggest that TKI therapy in such patients who develop LMD might extend survival [8, 110–112]. CNS penetration of these agents at standard doses has been found to be low, with improved penetration and concentration of gefetinib versus erlotinib [113]. Initial studies of higher dose therapy of erlotinib and gefetinib have shown promising improvements in neurological symptoms, although unacceptable toxicity has been described with 150–200 mg daily erlotinib [114–116]. However, early case series suggest that pulsatile erlotinib at high dosages can achieve increased penetration and efficacy while minimizing toxicity in patients who have progressed on standard TKI therapy [117]. Afatinib, a second generation TKI, has been shown to improve intrathecal penetration, improve survival in patients with CNS metastases, and elicit cerebral response in patients with LMD [118, 119]. Initial studies have also shown third generation TKIs, such as osimertinib (AZD9291) and AZD3759, may also be effective in treating TKI-resistant LMD from mutated EGFR lung cancer [120, 121].

Tesevatinib (XL647) is an oral TKI that can cross the blood-brain barrier and deposit in the leptomeninges. In an ongoing multicenter Phase II study (NCT02616393), patients with LMD secondary to EGFR-mutated NSCLC in one arm receive 300 mg of tesevatinib once daily. The primary outcome measure is improvement in clinical activity based on Common Terminology Criteria for Adverse Events (CTCAE v4.03).

Similarly, NSCLC with anaplastic lymphoma kinase (ALK) translocations may be effectively treated by ALK inhibitors such as crizotinib [122, 123]. However, patients with LMD remain poorly treated due to poor CNS penetration and subsequent reseeding of metastases [16, 124, 125]. Initial studies of second generation ALK inhibitors, such as ceretinib and alectinib, have shown increased CNS penetration, decreased radiological burden of metastases and LMD, and improvement in symptoms [126–131].

Ceritinib is currently being studied in multiple Phase II and III studies of LMD secondary to NSCLC with documented ALK rearrangement. Phase II trials include ASCEND-2, which treats crizotinib-resistent LMD, and ASCEND-3, which treats ALK-inhibitor naive LMD. Phase III trials include the ASCEND4 trial, where treatment is randomized to platinum pemetrexed versus ceritinib, and the ASCEND5 trial, where patients failing crizotinib are randomized to pemetrexed, docetaxel, or ceretinib [131]. To date, the ASCEND-2 and ASCEND-3 trial showed improved whole-body overall response rate (ASCEND-2: 38.6%, ASCEND-3: 58.9%) and progression free survival (ASCEND-2: 5.7 months, ASCEND-3: 11.1 months).

### Trastuzumab

Trastuzumab is a highly successful targeted monoclonal antibody therapy for HER2 positive cancers, but systemic administration can leave brain metastases and LMD as sanctuary sites [132–135]. Although pooled analysis of 17 patients with LMD from HER2 positive breast cancer suggested that trastuzumab may improve disease progression, randomized clinical trials are lacking [136]. In an ongoing Phase I and II trial, 34 adult patients were grouped into four cohorts of 3–6, with each cohort receiving varying dosages of IT trastuzumab twice a week for four weeks, then weekly for four weeks, and then every two weeks. Clinical, radiological, and CSF cytological responses are evaluated. Initial results from the Phase I study suggested increased efficacy at high dosages (80 mg), with which the Phase II study is now progressing [137].

### Immunotherapy

Immunotherapies have emerged as promising therapeutic options for several types of cancer including primary malignancies of the CNS and brain metastases. However, data are very limited on the utilization of these treatment modalities in patients with LMD [138]. There are a few studies reporting on either immune checkpoint blockade, intrathecal interleukin 2 (IL-2), or intrathecal tumor-infiltrating lymphocyte (TIL) therapies.

Checkpoint inhibitors such as ipilimumab and nivolumab, monoclonal antibodies that blocks CTLA-4 and PD-1, respectively, remain poorly studied in LMD, despite success and FDA approvals for a number of solid tumors. One case report documents a patient with melanoma-associated LMD who received WBRT followed by ipilimumab and survived for more than 18 months [139]. On the other hand, there is a case report of radiographic LMD mimicry in a separate melanoma patient treated with ipilimumab [140]. Regarding nivolumab, one study demonstrated improvement in neurological symptoms (auditory hallucinations) in an advanced NSCLC patient with LMD after treatment administration. Furthermore, the patient went on to exhibit a 7-month-progression-free survival [141]. Ultimately, further study of these agents is warranted.

Limited results are also reported for intrathecal IL-2 and TIL therapies. In a study of 42 patients with melanoma-associated LMD who were treated with intrathecal IL-2, median survival was found to be 9.1 months (range 0.7–86.2) with 16% of patients surviving more than 24 months [142]. These are certainly promising results, when compared to the normal median survival of 10 weeks in patients with metastatic melanoma LMD [143]. Additionally, a case report revealed radiographic disease stabilization in a patient with LMD from metastatic melanoma after administration of intrathecal autologous TILs in combination with intrathecal IL-2. Unfortunately, the regimen did not control the parenchymal brain metastases, which progressed 3 months following therapy [144]. Although such findings are encouraging, further well-designed randomized controlled studies are needed to evaluate the efficacy of immunotherapies in patients with LMD.

## FUTURE DIRECTIONS

Understanding the biology of LMD has created outlets for new molecular diagnostic and therapeutic strategies. Making use of these techniques will provide mechanisms for earlier detection and more effective treatments.

### Diagnostics

Looking for CSF biomarkers with high sensitivity can be more effective for early diagnosis of LMD as compared to CSF cytology and MRI. The latter both require a substantial level of disease progression in order to detect abnormalities, thereby contributing to delay in the treatment process. Treatment delay due to the insensitivity of current diagnostic methods can be improved through the analysis of CSF biomarkers. Testing for CSF VEGF has been shown to exhibit 75% sensitivity, 97% specificity, and 94% negative predictive value in breast cancer LMD diagnosis [46]. Levels of LMD-derived cell-free DNA (cfDNA), which can be low in plasma due to the BBB, can also be analyzed in CSF to follow both progression and development of drug-resistance mutations, and thereby guide therapy [145]. For example, a recent study by Zhao *et al.* of seven patients with LMD secondary to EGFR-positive TKI-resistent NSCLC patients found that a majority of patients were EGFR sensitive in CSF but not in plasma, possibly indicating poor TKI CSF penetration [146].

MicroRNA studies are able to detect abnormal levels of cancer-associated microRNAs in the CSF. In patients with metastatic brain and lung cancer, CSF miR-10b and miR-21levels increased significantly compared to tumors in remission and other non-neoplastic conditions. Using longitudinal microRNA profiles, disease activity and treatment response can be monitored more effectively in patients with NSCLC metastases [147]. Furthermore, proteomic analysis of CSF can be used to identify peptides expressed in patients with LMD. The MALDI peptide analysis of CSF specifically is able to identify several proteins involved in the host-disease interaction, inflammation, and immunity typically seen in neoplastic processes [148].

Circulating tumor cells (CTC) in CSF provide another option for early detection of LMD. Using this method, molecular tumor cell markers, such as epithelial cell adhesion molecule (EPCAM), can be used to identify metastatic cells in the CSF for diagnosis. Additionally, CTC methods enable the capture of live tumor cells, which can contribute to the current understanding of LMD biology [149].

### Treatments

Today, LMD is considered a fatal complication of cancer, as survival following diagnosis is devastatingly short. As science continues to reveal the underlying biology of LMD, treatments that reach the meninges and CSF while targeting the relevant molecular markers is essential to making advancements in diagnosis and treatment. Because most patients do not typically die solely from LMD, it is important for future therapies to address the systemic cancer in addition to the metastatic disease to improve survival. Progress is being made through the use of combination therapies, but there is still a large clinical unmet need for innovative treatments following diagnosis.

## Abbreviations

ALK: Anaplastic lymphoma kinase
ALL: Acute lymphoblastic leukemia
Ara-C: Cytarabine
CEA: Carcinoembryonic antigen
CI: Confidence interval
CNS: Central nervous system
CSF: Cerebrospinal fluid
CTC: Circulating tumor cells
DHFR: Dihydrofolate reductase
EGFR: Epidermal Growth Factor Receptor
EPCAM: Epithelial cell adhesion molecule
HR: Hazard ratio
IL: Interleukin
KPS: Karnofsky Performance Score
LMD: Leptomeningeal disease
miR: micro-ribonucleic acid
MRI: Magnetic resonance imaging
MTX: Methotrexate
NHL: Non-Hodgkin's lymphoma
NSCLC: Non-small-cell lung cancer
RANO: Response Assessment in Neuro-Oncology group
TIL: Tumor-infiltrating lymphocyte
TKI: Tyrosine kinase inhibitor
TMZ: Temozolomide
VEGF: Vascular endothelial growth factor
WBRT: Whole brain radiation therapy
